# Psychometric properties of the expanded prostate cancer index composite - 26 instrument in a cohort of radical prostatectomy patients: theoretical and practical examinations

**DOI:** 10.1186/s12894-017-0302-7

**Published:** 2017-12-02

**Authors:** Karol Axcrona, Rasmus Nilsson, Bjørn Brennhovd, Øystein Sørebø, Sophie D. Fosså, Alv A. Dahl

**Affiliations:** 1 Department of Urology, Akershus University Hospital, Lørenskog, Norway; 2 Department of Urology, Telemark Hospital, Skien, Norway; 3 Department of Urology, Oslo University Hospital, The Norwegian Radium Hospital, Oslo, Norway; 4 School of Business and Social Sciences, University College of Southeast Norway, Hønefoss, Norway; 5 National Resource Center for Late Effects after Cancer Treatment, Oslo University Hospital, The Norwegian Radium Hospital, P.O. Box 4453, Nydalen, 0424 Oslo, Norway; 6 Faculty of Medicine, University of Oslo, Oslo, Norway

**Keywords:** Prostate cancer, Adverse effects, Patient-reported outcome measures, The expanded prostate cancer index composite 26 item version, The UCLA prostate cancer index

## Abstract

**Background:**

Recently, the Expanded Prostate Cancer Index Composite 26-item version (EPIC-26) was recommended for the assessment of adverse effects after the treatment of prostate cancer without clear reasons. This decision encouraged us to review the questionnaire development from the UCLA Prostate Cancer Index (UCLA-PCI) to the EPIC-16 CP with a focus on psychometric properties. We also reviewed PubMed for papers concerning such properties of the EPIC-26 since 2012 (latest review in 2011). Finally, we examined the psychometric properties of the EPIC-26 in a sample of Norwegian males treated with robot-assisted laparoscopic prostatectomy (RALP).

**Methods:**

This study used three methods: (1) Comparison of the content of the UCLA-PCI, EPIC-50, EPIC-26, and EPIC-16 CP; (2) Review of EPIC-26 and EPIC-16 CP papers in PubMed from 2012 to 2016, identifying papers reporting on the psychometric properties of these questionnaires; and (3) Psychometric examination of the EPIC-26 rating in 651 Norwegian men treated with RALP at a mean of 3.2 years post-surgery.

**Results:**

The questionnaire development showed a significant increase in bother versus function items, and the EPIC-26 contains eight function and 18 bother items. Twelve papers concerning the EPIC-26 available on PubMed since 2012 support the psychometric properties of the EPIC-26. The Norwegian EPIC-26 findings supported the psychometric properties of the EPIC-26, but suggested six subdomains both by exploratory and confirmatory factor analyses.

**Conclusions:**

In general our examinations supported the adequate psychometric properties of the EPIC-26, although the factor structure, construct and predictive validity of the instrument should be examined further.

## Background

There is an increasing understanding within the uro-oncological community that the endpoints of prostate cancer (PCa) treatment should not only reflect disease recurrence and survival but also include patient-reported outcome measures (PROMs). In particular, PCa is a disease wherein the health-related quality of life (HRQoL)-related issues are crucial, since most patients live with the malignancy for many years. A major point of discussion regarding the introduction of PCa screening has been the diminished HRQoL affecting patients because of the diagnosis itself or of radical treatment for the disease [1, 2].

The development and increased use of PROMs for PCa have proliferated for many years, and this has continued in recent years. In addition, most physician-reported outcome measures show discrepancies with the patients’ experience of adverse effects after treatment [3]. PROMs are covered by questionnaires, which should document sufficient psychometric properties in their development process, including reliability and validity. In addition, PROMs should be founded on pre-diagnostic levels in a relevant population for such adverse effects, and should cover adverse effects that significantly affect the patients’ HRQoL. The validity of PROMs is based on continuous development over time reflecting improvements in treatment techniques, health-care delivery, and the changing priorities of society [4]. Importantly, PROMs also seem to be dependent on language and the cultural background of the samples studied [5]. Hamoen et al. [6] give a concentrated overview of psychometric key concepts for urologists and uro-oncologists, and the concepts relevant for this paper are given in Table 1.

**Table 1:** Explanations of key psychometric concepts described in this paper

Explanatory factor analysis	Statistical exploration of the underlying number of factors included in a questionnaire. The factor structure is the described by the number of factors, the items’ loading and the overall explained variance
Confirmatory factor analysis	Statistical analysis testing whether the data fit a hypothesized measurement model of the questionnaire. Indicators of adequate model fit are given in the text.
Feasibility	Indicates whether test persons find the questionnaire easy or complicated to complete.
Internal consistency	Describes how well subscale items of the questionnaire go together, and eventually subscales in relation to the total questionnaire.
Criterion validity	The questionnaire’s correlation with ‘gold standard’ questionnaires for the same concepts.
Convergent validity	High correlations with other questionnaires covering the same concepts.
Divergent validity	Low correlations with questionnaires covering other concepts.
Construct validity	How well the questionnaire corresponds to other ways of measuring the construct i.e. various adverse effects.
Predictive validity	The ability of the questionnaire score to predict important future outcome.
Responsiveness to change	The ability of the questionnaire to identify clinical changes in the domains covered by the questionnaire.

Recently, two international working groups recommended the Expanded Prostate Cancer Index Composite 26-question short form (EPIC-26) [7] as a Standard Set of Patient-centered Outcome for men with both localized [8] and advanced PCa [9]. Interestingly, the groups hardly provided any reasons for their choice, except indirectly stating that the EPIC-26 covered post-radiation rectal bleeding and therefore was preferred over the 16 items Expanded Prostate Cancer Index Composite for Clinical Practice (EPIC-16 CP) [10]. In a review by Rnic et al. [11] analyzing the psychometric properties of 29 PROMs for localized PCa, the EPIC-26 was rated among the top three instruments together with the UCLA Prostate Cancer Index (UCLA-PCI) [12], while the EPIC-50 [13] got lower ratings. Hamoen et al. [6] published a more extensive psychometric evaluation of 20 PROMs for HRQoL in PCa patients including the UCLA-PCI and the EPIC-50, and they recommended the UCLA-PCI, which according to them had been used in 268 studies of 135,366 patients. They also pointed out the problem of making recommendations when one instrument was an extended version of another, such as the EPIC-50 in relation to the UCLA-PCI. Comparisons were also considered more difficult since not all psychometric properties were tested in the validation studies of these two instruments. The same problem of comparison also concerned the reduced versions of the EPIC-26 and the EPIC-16 CP based on the EPIC-50. Using the Evaluating Measures of Patient-Reported Outcomes examining eight instruments, Schmidt et al. [14] recommended the EPIC-50 versus the UCLA-PCI due to a better overall methodology score. Considering these three psychometric reviews, the abovementioned international recommendation [8, 9] of the EPIC-26 appeared problematic, particularly in the absence of a detailed rationale supporting the recommendation.

## Methods

The current paper examines three issues relevant for the validity of the EPIC-26 recommendation: (1) The developmental history of the UCLA-PCI and EPIC instruments; (2) Review of psychometric studies of the EPIC-26 published since 2012; and (3) Psychometric testing of the EPIC-26 in a Norwegian sample of PCa patients treated by robot-assisted prostatectomy (RALP) at a median of 3 years post-surgery.

## Results

### Development of the UCLA-PCI and the EPIC PROMs (Table 2)

#### The UCLA-PCI

The American urologist Mark S. Litwin and the CaPSURE-group have led the development and psychometric testing of PCa-relevant PROMs. They first developed the UCLA Prostate Cancer Index (UCLA-PCI) [12] that included 20 items covering both function and bother items (experienced problems) within the urinary, bowel, and sexual domains, with 17 items covering function (85% of all items) and 3 items covering bother, one for each of the domains (15% of all items). The item distribution is shown in Table 2.

**Table 2:** Item distribution of the UCLA-PCI and EPIC PROMs

Items	UCLA-PCI	EPIC 50	EPIC-26	EPIC-16 CP
Urinary domain				
Function				
Leakage	1	1	1	0
Control	1	1	1	1
Pad use	1	1	1	1
Hematuria	0	1	0	0
Pain or burning	0	1	0	0
Bother				
Urinary symptom bother	2	6	5	4
Overall urinary bother	1	1	1	1
Bowel domain				
Function				
Rectal urgency	1	1	0	0
Loose or liquid stools	1	0	0	0
Uncontrolled leakage	0	1	0	0
Bloody stools	0	1	0	0
Painful movements	0	1	0	0
Frequency of movements	0	1	0	0
Cramping pain	1	1	0	0
Bother				
Bowel symptom bother	1	6	5	2
Overall bowel bother	1	1	1	1
Sexual domain				
Function				
Sexual desire	1	1	0	0
Ability to have erections	1	1	1	0
Ability to reach orgasm	1	1	1	1
Quality of erections	1	1	1	1
Frequency of erection	1	1	1	0
Awakening with erection	1	1	0	0
Overall sexual function	0	1	1	0
Any sexual activity	0	1	0	0
Had intercourse	1	1	0	0
Bother				
Sexual symptom bother	0	3	0	0
Overall sexual bother	1	1	1	1
Hormonal domain				
Function				
Hot flashes	0	1	0	0
Breast tenderness	0	1	0	0
Depression	0	1	0	0
Lack of energy	0	1	0	0
Weight change	0	1	0	0
Bother				
Hormonal side effects bother	0	6	5	3
Overall hormonal bother	0	0	0	0

As demonstrated by the reviews [6, 11, 14], the psychometric documentation of the UCLA-PCI is extensive. All the three reviews recommend the UCLA-PCI as compared to other PROMs for PCa. The main criticism has been poor coverage of irritative voiding symptoms and lacking coverage of adverse effects related to neo-adjuvant or adjuvant androgen deprivation therapy (ADT) [11].

#### The EPIC-50

The Expanded Prostate Cancer Index Composite (EPIC-50) was developed since the UCLA-PCI addressed neither irritative and obstructive voiding symptoms nor specific symptoms related to ADT. However, the main expansion concerned additional bother items corresponding to each of the symptom items, without any reasons given for their introduction [13]. The three overall bother items of the UCLA-PCI were kept, while no corresponding item for the hormonal domain was included. The item distribution of the EPIC-50 is displayed in Table 2.

Compared to the 20 items of the UCLA-PCI, the EPIC-50 represented a considerable expansion of items and, accordingly, longer completion time for the patients. The balance between function and bother items was changed since 48% of the items now concerned bother. Most items’ responses offered rating alternatives from worst (bad) to best (good). The item on the frequency of bowel movements (item #18), however, had no best or worst alternatives, and concerning the sexual symptom items, the best and worst alternatives were not stated explicitly. The relations between the overall and function bother scores were nowhere specified. For the urinary incontinence and irritation/obstruction subscales, the function and bother items were combined without any explanation. Since ≥40% of the sample scored maximum on the urinary function and irritation/obstruction subscales, a problem with ceiling effects could be relevant [13]. In spite of these weaknesses, all the three reviews [6, 11, 14] recommended the EPIC-50 in comparison with other relevant PCa PROMs.

#### The EPIC-26

The development of the EPIC-26 was motivated by a presumed better clinical utility of a shortened version of the EPIC-50 [7]. The main procedure for item reduction was item-scale correlations geared by reliability rather than validity considerations. The items distribution is shown in Table 2. With 8 function items and 18 symptom bother and overall bother items, the proportion of bother items increased to 69% in the EPIC-26. The EPIC-26 included 12 (60%) of the original 20 UCLA-PCI items [7]. The EPIC-26 was the top recommendation of the review by Rnic et al. [11], while the two other reviews did not include the EPIC-26.

#### The EPIC-16 CP

Inspired by successful PROMs measuring lower urinary tract symptoms and erectile dysfunction as well as given the need for improving weaknesses of the EPIC-26, the EPIC-16 CP was developed [10]. With 4 function items and 12 symptom bother and overall bother items, the proportion of bother items increased to 75% in the EPIC-16 CP. The item distribution is shown in Table 2.

### Review of recent psychometric studies of the EPIC-26 (Table 3)

The psychometric properties of the EPIC-50 and the UCLA-PCI have been reviewed recently [6, 14]. Concerning the psychometric review of the EPIC-26, Rnic et al. [11] included papers published before the end of 2011. Therefore, in March 2016, we performed a PubMed search with the term “Expanded Prostate Cancer Index Composite” retrieving 266 papers; of these, 161 of them were published in 2012 or later and therefore were not included in the review by Rnic et al. Two of the authors (RN and AAD) read the 161 abstracts, of which 22 full papers were examined. Among them, 13 papers included psychometric data on the EPIC-26 (Table 3). Only the paper by Chipman et al. [15] contained psychometric data on the EPIC-16 CP.

**Table 3:** Studies examining the psychometric properties of the EPIC-26 since 2012

Study (reference)	Psychometric findings
Korzeniowski et al. 2016 [16]	Informative and useful for patient communication as judged by clinicians
Sharma et al. 2016 [17]	Good feasibility both on paper and electronically
Skolarus et al. 2012 [18]	Good feasibility both on paper and automatic telephone response
Sampurno et al. 2015 [19]	Good feasibility both on paper and interactive voice method
Fosså et al. 2016 [20]	Internal consistencies alpha 0.64–0.91 of the 5 domains
Skolarus et al. 2012 [18]	Good test-retest reliability
Sampurno et al. 2015 [19]	Good test-retest reliability
Ellison et al. 2013 [21]	Criterion validity with the Incontinence Severity Index
Fosså et al. 2016 [20]	Criterion validity with the International Prostate Symptom Score
Punnen et al. 2013 [22]	Convergent validity of urinary and sexual bother scores and Generalized Anxiety Disorders Screener (GAD-7) and Distress Thermometer, and sexual bother with Patient Health Questionnaire-9 (depression)
Evans et al. 2015 [23]	Convergent validity with the SF-12 (quality of life)
Watson et al. 2015 [24]	Convergent validity of urine and bowel domains with health (EQ-5D-5 L), unmet needs (SCNS-SF34), anxiety/depression (HADS), and self-efficacy (Cancer Survivors Self Efficacy Scale)
Schofield et al. 2012 [25]	Divergent validity with unmet needs (SCNF-SF 34)
Evans et al. 2015 [23]	Predictive validity with the SF-12 (quality of life)
Recklitis et al. 2014 [26]	Predictive validity of hormonal subscale score and more suicidal ideation
Evans et al. 2015 [23]	Responsiveness to change (minimally important differences)
Skolarus et al. 2015 [27]	Responsiveness to change (minimally important differences)
Tavlarides et al. 2015 [28]	Responsiveness to change documented
Fosså et al. 2016 [20]	Responsiveness to change documented

Our review confirmed the good feasibility, internal consistencies, and test-retest reliability previously demonstrated for the EPIC-26 [16–26]. In addition, responsiveness to change over time was amply demonstrated [20, 23, 27, 28]. The convergent, divergent, and criterion validity of the EPIC-26 were also supported [22–24]. However, the stronger types of construct and predictive validity were hardly covered. Predictive validity with future HRQoL (the Short Form 12) was documented by Evans et al. [23]. Interestingly, the hormonal subscale of the EPIC-26 predicted future suicidal ideation [26].

### Psychometric examination of the EPIC-26 in a Norwegian sample

The database for our psychometric testing comprised the complete EPIC-26 ratings from 651 men operated with robot-assisted laparoscopic prostatectomy for PCa at the Oslo University Hospital, The Norwegian Radium Hospital, between January 1, 2005 and July 31, 2010 [29, 30]. Initially, 982 primary operated men were invited to a cross-sectional, mailed questionnaire study in March 2011, and 777 of them responded (79% response rate) at a median of 2.9 (range, 0.5–6.1) years postoperatively. However, only 651 (83%) had completed all items of the EPIC-26, and this sample was used for our psychometric analyses. Among them, 142 patients (22%) self-reported relapse, and 104 patients described that they had been treated with radiotherapy and 53 with ADT. The questionnaire also included the Hospital Anxiety and Depression Scale (HADS) [31], the Short Form-12 (SF-12) Measuring HRQoL [32], and a 6-item scale examining the personality trait of neuroticism [33].

The following statistical analyses were performed on our EPIC-26 sample: internal consistencies with Cronbach’s coefficient alpha and correlation coefficients with Spearman’s coefficient rho. Explained variance was the second power of the correlation coefficient. Exploratory factor analysis (EFA) was performed as principal component analysis with Oblimin rotation with Kaiser normalization including EPIC-26 items with a factor loading of ≥0.30. Confirmatory factor analysis (CFA) was done with LISREL analyses (i.e., Maximum Likelihood estimation). The fit of the CFA model was evaluated through examination of the sizes of the factor loadings and values for the fit indices. Acceptable fit was shown by Root Mean Square Error of Approximation <0.07; Standardized Root Mean Square Residual <0.08; The Comparative Fit Index ≥0.95 and a Parsimonious Normed Fit Index > .50 [34, 35]. There is, however, no exact threshold for PNFI in the literature but a value above .50 indicates a parsimonious model. In addition to these fit indices, we also included the Akaike Information Criterion with the purpose to compare alternative measurement models.

Satisfactory Cronbach’s coefficients alphas were observed for all EPIC-26 domain scales (Table 4). The proportion of scores with floor effects was satisfactory; however, 62.5% of the men showed ceiling effects on the bowel subscale (Table 4).

**Table 4:** A-C Norwegian sample findings on EPIC-26 (*N* = 651)

A. Characteristics of EPIC-26 domain-specific scores					
Domain	Mean (SD)	% Minimum	% Maximum	Median (range)	Cronbach’s α
Urinary					
Incontinence	71.8 (27.8)	1.5	27.9	79.3 (0.0–100.0)	0.89
Irritation	86.7 (15.1)	0.0	29.8	90.0 (10.0–100.0)	0.72
Bowel	93.7 (12.5)	0.0	62.5	100.0 (12.5–100.0)	0.82
Sexual	32.9 (27.7)	11.8	1.1	23.7 (0.0–100.0)	0.78
Hormonal	86.9 (17.5)	0.3	41.8	95.0 (0.3–100.0)	0.78
B. Correlation matrix of EPIC-26 domains and psychosocial scales (*p < 0.001)					
EPIC domains	HADS-A	HADS-D	SF-12 PCS	SF-12 MCS	Neuroticism
Urinary					
Incontinence	−0.16*	−0.17*	0.27*	0.13*	−0.22*
Irritation	−0.24*	−0.26*	0.34*	0.21*	−0.30*
Bowel	−0.27*	−0.26*	0.28*	0.20*	−0.26*
Sexual	−0.06	−0.16*	0.15*	0.11*	−0.16*
Hormonal	−0.48*	−0.53*	0.47*	0.58*	−0.66*
Urinary bother	−0.17*	−0.19*	0.29*	0.20*	−0.26*
Bowel bother	−0.23*	−0.21*	0.24*	0.15*	−0.21*
Sexual bother	−0.14*	−0.13*	0.15*	0.15*	−0.18*
C. Correlation matrix of EPIC-26 domains and prostate cancer variables (*p < 0.02)					
EPIC-26 domains	D’Amico risk score	Positive margins	Prostate volume	Nerve-sparing	Relapse
Urinary					
Incontinence	−0.14*	−0.03	−0.12*	0.16*	−0.08
Irritation	−0.13*	−0.07	−0.02	0.15*	−0.13*
Bowel	−0.11*	−0.07	−0.02	0.09*	−0.06
Sexual	−0.19*	−0.08	−0.19*	0.28*	−0.10*
Hormonal	−0.10*	−0.08	0.04	0.16*	−0.20*
Urinary bother	−0.11*	−0.05	−0.06	0.11*	−0.13*
Bowel bother	−0.11*	−0.10*	−0.03	0.09*	−0.05
Sexual bother	−0.10*	−0.13*	−0.16*	0.16*	−0.02

All EPIC-26 domain scales, except for the hormonal one, showed satisfactory discriminant validity in relation to the HADS anxiety and depression, the SF-12 PCS and MCS, and neuroticism scores (Table 4B). In contrast, testing the EPIC-26 domain scales in relation to major PCa outcome characteristics that were registered at baseline, demonstrated small correlation coefficients (Table 4C).

The EFA showed an explained variance of 67% for a six factor solution (Table 5). The bowel and sexual domain scales of the EPIC-26 were supported (factors 1 and 3), while the urinary and hormonal domain scales showed two factors each (1 and 6 versus 4 and 5), respectively. The urinary factors corresponded to the incontinence and irritation/obstruction subscales described for the EPIC-26 [7], while no such correspondence was shown for the two factors of the hormonal domain.

**Table 5:** Explorative principal component analysis with direct oblimin rotation of the EPIC-26 scores of the Norwegian sample (*N* = 651)

EPIC-26 items	Factors					
	1	2	3	4	5	6
1. Leaked urine	**0.90**					
2. Urinary control	**0.86**					
3. Number of pads	**0.89**					
4 A. Dripping or leaking of urine	**0.92**					
B. Pain or burning on urination						**0.89**
C. Bleeding with urination						**0.88**
D. Weak urine stream or incomplete emptying						**0.44**
E. Need to urinate frequently	**0.42**					
5. Overall problem with urinary function	**0.81**					
6 A. Bowel urgency		**0.82**				
B. More frequent bowel movements		**0.84**				
C. Loosing control over stools		**0.77**				
D. Bloody stools		**0.48**				
E. Abdominal/pelvic/rectal pain		**0.50**				
7. Overall problem with bowel habits		**0.87**				
8 A. Ability to have an erection			**0.93**			
B. Ability to reach orgasm			**0.76**			
9. Quality of erections			**0.91**			
10. Frequency of erections			**0.91**			
11. Ability to function sexually			**0.91**			
12. Overall problem with sexual function			0.39		0.40	
13 A. Hot flashes				**0.67**		
B. Breast tenderness/enlargement				**0.78**		
C. Feeling depressed					**0.82**	
D. Lack of energy					**0.81**	
E. Change in body weight				**0.56**	0.41	
Per cent explained variance	26.7	10.2	13.5	7.1	5.3	4.2

Coefficients below .30 were supressed

Bold texts represent factor loading > 0.40

Item 12 showed a factor loading (factor 3) and a cross-loading (factor 5) that were approximately equal, while item 13E showed up with a substantial cross loading (factor 4 and 5). The two identified cross loadings indicate that there may be discriminant validity problem on the item level regarding items 12 and 13E. We, however, decided to test the EFA generated six-factor solution with CFA before we conclude on the issue of discriminant validity.

**Fig. 1:**
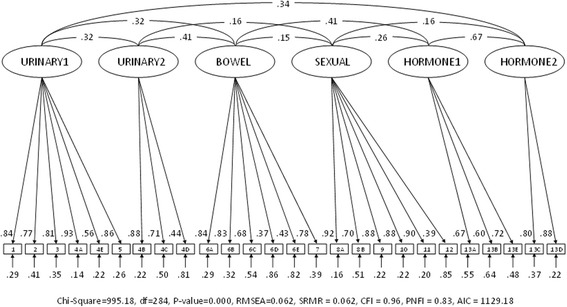
Confirmatory factor analysis of the 6-factor EPIC-26 in the Norwegian sample (*N* = 651)

As demonstrated in Fig. 1, item 12 showed a relatively low factor loading (0.39) in the CFA while item 13E showed an acceptable loading (0.72). Item 12 seems not to fit well with the EPIC-26 measurement model. The wording on item 12 deals with an “overall problem with sexual function” which indicates a global wording that is less specific than the other items of the EPIC-26. Hence, this may explain the low factor loading and the identified problem with discriminant validity.

**Fig. 2:**
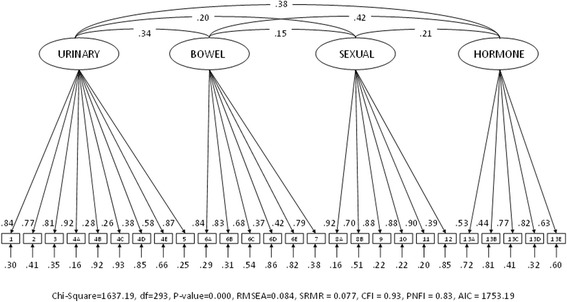
Confirmatory factor analysis of the 4-factor EPIC-26 in the Norwegian sample (*N* = 651)

The CFA showed adequate fit values for the six-factor solution (Fig. 1 and Table 6). The four-factor solution, however, showed some fit values that were outside the threshold values (Fig. 2 and Table 6).

**Table 6:** Measurement model fit for EPIC-26 scores of the Norwegian sample (*N* = 651)

Fit-indexes	Six-factor solution	Four-factor solution	Threshold values
Chi-Square	995.18	1637.19	–
df	284	293	–
*P*-value	0.000	0.000	–
*RMSEA* (95% CI)	0.062 (0.058;0.066)	0.084 (0.080;0.088)	0.07
*SRMR*	0.062	0.077	0.08
*CFI*	0.96	0.93	0.95
*PNFI*	0.83	0.83	0.50
*AIC*	1129.18	1753.19	Lowest value are optimal

## Discussion

### Development of the UCLA-PCI and the EPIC PROMs

In 1994, Litwin was inspired by the urinary questionnaire from the Olmsted County studies, in which 12 items concerning functions and symptoms had 12 corresponding bother items [36]. Accordingly, Litwin stated that PCa-related PROMs must cover both function and bother: “Function and bother must be measured, and the distinction between these two dimensions is important to recognize. Some men with significant sexual dysfunction were minimally bothered, while others with only mild dysfunction may be miserable… Therefore, while sexual function and bother are undeniably linked, they are independent domains and must be measured separately ([37], page 1884).” In the light of this statement, 15% bother items in the UCLA-PCI seem a small proportion which is compensated by 48% bother items in the EPIC-50. This equality was then changed over again with 69% bother items in the EPIC-26 and 75% of such items in the EPIC-16 CP. During the development of these PROMs, the proportion of bother items increased at the cost of functional items (Table 2). However, the instrument developers did not explain this conceptual change towards reduced interest in the functional results of the patients. From a clinical point of view, Litwin’s original statement of equal relevance of function and bother seems to be reasonable, and it is difficult to gauge the reasons for the increased focus on bother problems in the later instruments. In addition, the measurement of functional outcomes enables a more objective assessment of the effectiveness of treatment modifications.

### Review of recent psychometric studies of the EPIC-26

The PubMed update from 2012 on the EPIC-26 showed considerable popularity of this PROM, but only 8% (13/161 publications) contained psychometric data (Table 3). The update confirmed the good feasibility, internal consistencies, and test-retest reliability and responsiveness to change of the EPIC-26. The reliability of the EPIC-26 has been further supported, although factor analytic studies remain infrequent.

The demonstration of various types of validity is a continually ongoing process [4]. *Content validity* concerns the extent to which the EPIC-26 adequately covers the expectable adverse effects related to the treatment of PCa. For example, the UCLA-PCI lacked some content validity since that PROM omitted the hormonal domain included in the EPIC-26. On the other hand, the focus on bother rather than function could represent a weakened content validity of the EPIC-26 as compared to the EPIC-50.

*Convergent validity* confirms that the domains of the EPIC correlate highly with other established PROMs covering the same domains. Correspondingly, *divergent validity* relates to low correlation with established PROMs measuring unrelated concepts. In our update, we found support for both these types of validity in relation to the EPIC-26 [20–23]. *Criterion validity* (which includes convergent and divergent validity) involves the assessment of the EPIC-26 against more well-established questionnaires covering all or some of the same treatment-related domains. Both the previous review of Rnic et al. [11] and our update (references #18 and 19) supported such validity of the EPIC-26.

*Construct validity* concerns the relationship of the EPIC-26 to theoretical constructs about adverse effects related to the treatment modalities of PCa. A problem in this regard is the construction of the EPIC-26 as a multidimensional PROM covering adverse effects related to surgery, radiotherapy, and hormonal treatment. One construct is erectile dysfunction related to nerve damage during prostatectomy or radiotherapy, while another is proctitis after radiotherapy. We must therefore question whether construct validity is a meaningful concept for the total EPIC-26, or that such validity only can be studied for each of its domains (subscales). For example, many studies have examined the relationship between nerve sparing and post-surgical erectile dysfunction and found a considerable correlation [38], which supported the construct validity of the sexual domain of the EPIC-26. We conclude that future studies of the construct validity of the EPIC-26 on the domain level should be performed.

*Predictive validity* concerns the ability of the EPIC-26 to predict future health status, test results, or events in PCa patients. Urologists and uro-oncologists are familiar with this concept from the D’Amico risk index based on the pre-treatment prostate-specific antigen (PSA) level, Gleason score, and tumor stage [39] concerning biochemical outcomes for localized PCa. Additional prognostic factors include PSA velocity and doubling time (biochemical failure), BMI, primary Gleason score/grading system, the number of positive biopsy cores, and, in the case of radical prostatectomy, the presence of a positive surgical margin and the presence of perineural invasion [40, 41]. Baseline HRQoL scores also predicted PCa outcome in one study [42]. Thus far, the relationship between the EPIC-26 and these prognostic factors hardly has been studied.

Among the update studies, Evans et al. [23] observed that better baseline HRQoL scores predicted better EPIC-26 domain scores at 2 years post-treatment in a considerable sample treated with stereotactic body radiotherapy. Another update study found that suicidal ideation at a mean of 5 years after treatment was significantly predicted by the EPIC-26 hormonal domain score [26]. A reasonable explanation is that the hormonal items of depression, lack of energy, and changes in body weight are typical symptoms of a depressive disorder, which is a strong risk factor for suicidal ideation [43].

### Psychometric findings of the EPIC-26 in the Norwegian sample

Similar to the results of Szymanski et al. [7] and another recent study from Norway [20], we observed adequate internal consistencies (Cronbach’s coefficients alpha, 0.65–0.90) for the EPIC-26 domains (Table 4). Our mean domain scores and floor/ceiling proportions (Table 4) were both close to those observed in these previous studies [7, 20].

The present study confirmed the previous finding [24] that the EPIC-26 hormonal domain scores have a considerably strong correlation with anxiety/depression, HRQoL, and neuroticism, with explained variances ranging from 22% to 44% (Table 4B). With a maximum of 12% explained variance for the other EPIC-26 domain scores, discriminant validity in relation to such variables was confirmed.

The maximum explained variance of 8% for relevant predictors of PCa outcome variables at surgery and those concerning relapse indicated a lack of predictive validity for the EPIC-26 domain scores in this regard.

To the best of our knowledge, we have presented here the first factor analyses of the EPIC-26. In our sample, the optimal EFA solution identified six rather than the expected four factors showing the highest explained variance of 67%. This new solution was due to two factors (1and 6) within the urinary domain of the EPIC-26, defining items #4B (pain), 4C (bleeding with urination), and 4D (weak stream and incomplete emptying) as a second irritative urinary factor (Table 5). Correspondingly, two factors were identified within the hormonal domain (factors 4 and 5). Our EFA identified 2 EPIC-26 items with factor loading on two factors items #12 and 13E, that are in need of further investigation as relevant EPIC-26 items.

An EFA model of the EPIC-26 with 67% explained variance should be considered as reasonably good. Comparing fit values favors our six-factor solution (Table 6). There are, however, some issues that need to be considered before such a conclusion is drawn. The four-factor solution has some support in previous studies, and it appears as theoretically valid and parsimonious. The six-factor solution is based on an exploratory empirical approach (EFA) that may have generated a setting specific solution due to the composition of our sample. The EPIC instruments were meant to assess HRQOL after treatment for PCa by all modalities not just RP. A major limitation of our psychometric analyses is the absence of in our sample of patients who received radiation either alone or with ADT as their primary treatment.

## Conclusions

In this paper we have presented the development and psychometrics of the EPIC-26 and the problems associated with the recent recommendations of that questionnaire. The literature unanimously supports the reliability of the EPIC-26, although two items are of doubtful value in our study. Further studies of the EFA and CFA in other samples are needed. The content, discriminant and convergent validities of the EPIC-26 appear good, while the construct and predictive validities remain in need of further examination and development with different treatments.

## Abbreviations

CFA: Confirmatory factor analysis
EFA: Exploratory factor analysis
EPIC-16CP: Expanded Prostate Cancer Index Composite for Clinical Practice 16 items
EPIC-26: Expanded Prostate Cancer Index Composite 26 items
EPIC-50: Expanded Prostate Cancer Index Composite 50 items
HRQoL: Health-related quality of life
PCa: prostate cancer
PROMs: Patient-rated outcome measures
SF-12 MCS: Short Form 12: Mental composite scale
SF-12 PCS: Short Form 12: Physical composite scale
UCLA-PCI: University of California at Los Angeles Prostate Cancer Index

## References

[1] Schröder FH, Hugosson J, Roobol MJ, Tammela TL, Ciotto S, Nelen V (2009). Screening and prostate cancer mortality in a randomized European study. N Engl J Med.

[2] Schröder FH, Hugosson J, Roobol MJ, Tammela TL, Ciotto S, Nelen V (2012). Prostate cancer mortality at 11 years of follow-up. N Engl J Med.

[3] Steinsvik EA, Fosså SD, Axcrona K, Fransson P, Widmark A, Dahl AA (2010). Do perceptions of adverse events differ between patients and physicians? Findings from a randomized controlled trial of radical treatment of prostate cancer. J Urol.

[4] Fayers PM, Machin P (2007). Quality of life. The assessment, analysis and interpretation of patient-reported outcomes. 2nd ed.

[5] Storås AH, Sanda MG, Boronat OG, Chang P, Patil D, Crociani C (2016). Erectile dysfunction and sexual problems two to three years after prostatectomy among American, Norwegian, and Spanish patients. Clin Genitourin Cancer.

[6] Hamoen EH, De Rooij M, Witjes JA, Barentsz JO, Rovers MM. Measuring health-related quality of life in men with prostate cancer: A systematic review of the most used questionnaires and their validity. Urol Oncol. 2015;33(2) 69.e19–28 10.1016/j.urolonc.2013.10.005 24433753

[7] Szymanski KM, Wei JT, Dunn L, Sanda MG (2010). Development and validation of an abbreviated version of the expanded prostate index composite instrument for measuring health-related quality of life among prostate cancer survivors. Urology.

[8] Martin NE, Masey L, Stowell C, Bangma C, Briganti A, Bill-Axelson A (2015). Defining a standard set of patient-centered outcomes for men with localized prostate cancer. Eur Urol.

[9] Morgans AK, van Bommel AC, Stowell C, Abrahm JL, Basch E, Bekelmann JE (2015). Development of a standardized set of patient-centered outcomes for advanced prostate cancer: an international effort for a unified approach. Eur Urol.

[10] Chang P, Szymanski KM, Dunn RL, Chipman JJ, Litwin MS, Nguyen PL (2011). Expanded prostate cancer index composite for clinical practice: development and validation of a practical health related quality of life instrument for use in the routine clinical care of patients with prostate cancer. J Urol.

[11] Rnic K, Linden W, Tudor I, Pullmer R, Vodermaier A (2013). Measuring symptoms in localized prostate cancer: a systematic review of assessment instruments. Prost Cancer Prost Dis.

[12] Litwin MS, Hays RD, Fink A, Ganz PA, Leake B, Brook RH (1998). The UCLA prostate index: development, reliability, and validity of a health-related quality of life measure. Med Care.

[13] Wei JT, Dunn RL, Litwin MS, Sandler HM, Sanda MG (2000). Development and validation of the expanded prostate cancer index composite (EPIC) for comprehensive assessment of health-related quality of life in men with prostate cancer. Urology.

[14] Schmidt S, Garin O, Pardo Y, Valderas JM, Alonso J, Rebollo P (2014). Assessing quality of life in patients with prostate cancer: a systematic and standardized comparison of available instruments. Qual Life Res.

[15] Chipman JJ, Sanda MG, Dunn RL, Wei JT, Litwin MS, Crociani CM (2014). Measuring and predicting prostate cancer related quality of life changes using EPIC for clinical practice. J Urol.

[16] Korzeniowski M, Kalyvas M, Mahmud A, Shenfield C, Tong C, Zaza K (2016). Piloting prostate cancer patient-reported outcomes in clinical practice. Support Care Cancer.

[17] Sharma P, Dunn RL, Wei JT, Monite JE, Gilbert SM (2016). Evaluation of point-of-care PRO assessment in clinical settings: integration, parallel-forms reliability, and patient acceptability of electronic QOL measures during clinical visits. Qual Life Res.

[18] Skolarus TA, Holmes-Rovner M, Hawley ST, Dunn RL, Barr KL, Willard NR (2012). Monitoring quality of life among prostate cancer survivors: the feasibility of automated telephone assessment. Urology.

[19] Sampurno F, Ruseckaite R, Millar JL, Evans SM (2016). Comparison of patient-reported quality-of-life and complications in men with prostate cancer, between two modes of administration. Clin Genitourin Cancer.

[20] Fosså SD, Holck Storås A, Steinsvik EA, Myklebust TA, Eri LM, Loge JH (2016). Psychometric testing of the Norwegian version of the expanded prostate cancer index composite 26-item version (EPIC-26). Scand J Urol.

[21] Ellison JS, He C, Wood DP (2013). Stratification of postprostatectomy urinary function using expanded prostate cancer index composite. Urology.

[22] Punnen S, Cowan JE, Dunn LB, Shumay DM, Carroll PR, Cooperberg MRA (2013). Longitudinal study of anxiety, depression and distress as predictors of sexual and urinary quality of life in men with prostate cancer. BJU Int.

[23] Evans JR, Zhao S, Daignault S, Sanda MG, Michalski J, Sandler HM (2015). Patient-reported quality of life after stereotactic body radiotherapy (SBRT), intensity modulated radiotherapy (IMRT), and brachytherapy. Radiother Oncol.

[24] Watson E, Shinkins B, Frith E, Neal D, Hamdy F, Walter F (2016). Symptoms, unmet needs, psychosocial well-being and health status in survivors of prostate cancer: implications for redesigning follow-up. BJU Int.

[25] Schofield P, Gough K, Lotfi-Jam K, Aranda S (2012). Validation of the supportive care needs survey-short form 34 with a simplified response format in men with prostate cancer. Psycho-Oncologia.

[26] Recklitis CJ, Zhou ES, Zwerner EK, JC H, Kantoff PW (2014). Suicidal ideation in prostate cancer survivors: understanding the role of physical and psychological health outcomes. Cancer.

[27] Skolarus TA, Dunn RL, Sanda MG, Chang P, Greenfield TK, Litwin MS (2015). Minimally important difference for the expanded prostate cancer index composite short form. Urology.

[28] Tavlarides AM, Ames SC, Thiel DD, Diehl NN, Parker AS (2015). Baseline and follow-up association of the MAX-PC in men with newly diagnosed prostate cancer. Psycho-Oncologia.

[29] Brennhovd B, Axcrona K, Fosså SD, Giercksky KE, Vlatkovic L, Dahl AA (2013). Robot- assisted radical prostatectomy of clinical high-risk patients with prostate cancer: a controlled study of operative and short-term post-operative events. Scand J Urol.

[30] Østby-Deglum M, Brennhovd B, Axcrona K, Fosså SD, Dahl AAA (2015). Comparative study of erectile function and use of erectile aids in high-risk prostate cancer patients after robot-assisted laparoscopic prostatectomy. Scand J Urol.

[31] Bjelland I, Dahl AA, Haug TT, Neckelmann D (2002). The validity of the hospital anxiety depression scale. An updated literature review. J Psychosom Res.

[32] Gandek B, Ware JE, Aaronson NK, Apolone B, Bjorner JB, Bullinger M (1998). Cross-validation of item selection and scoring for the SF-12 Health Survey in nine countries: results from the IQOLA Project. International Quality of Life Assessment. J Clin Epidemiol.

[33] Grov EK, Fosså SD, Bremnes RM, Dahl O, Klepp O, Wist E (2009). The personality trait of neuroticism is strongly associated with long-term morbidity in testicular cancer survivors. Acta Oncol.

[34] Hooper D, Chen H (2012). Exploratory factor analysis. Approaches to quantitative research – theory and its practical application: a guide to dissertation students.

[35] Hu L, Bentler PM (1999). Cutoff criteria for fit indexes in covariance structure analysis: conventional criteria versus new alternatives. Struct Equat Model.

[36] Jacobsen SJ, Girman CJ, Guess HA, Panser LA, Chute CB, Oesterling JE (1993). Natural history of prostatism: factors associated discordance between frequency and bother of urinary symptoms. Urology.

[37] Litwin MS (1994). Measuring health related quality of life in men with prostate cancer. J Urol.

[38] Briganti A, Gallina A, Suardi N, Capitanio U, Tutolo M, Bianci M (2010). Predicting erectile function recovery after bilateral nerve sparing radical prostatectomy: a proposal of a novel preoperative risk stratification. J Sex Med.

[39] D’Amico AV, Whittington R, Malkowicz SB, Schultz D, Blank K, Broderick GA (1998). Biochemical outcome after radical prostatectomy, external beam therapy, or interstitial radiation therapy for clinically localized prostate cancer. JAMA.

[40] Crook J, Ots AF (2013). Prognostic factors for newly diagnosed prostate cancer and their role in treatment selection. Sem. Radiat Oncol.

[41] Martin NE, Mucci LA, Loda M, Depinho RA (2011). Prognostic determinants in prostate cancer. Cancer J.

[42] Braun DP, Gupta D, Staren ED (2012). Predicting survival in prostate cancer: the role of quality of life assessment. Support Care Cancer.

[43] American Psychiatric Association (2010). Practice guideline for the treatment of patients with major depressive disorder.

